# Neurophysiological monitoring of cranial nerves III and VI in endoscopic skull base surgery: scoping review and comparative study

**DOI:** 10.3389/fonc.2026.1831926

**Published:** 2026-09-14

**Authors:** Simona Serioli, Ginevra D’Onofrio, Federico Valeri, Alberto Pietrantoni, Laura Broglio, Ludovico Agostini, Pier Paolo Mattogno, Vito Stifano, Marco Maria Fontanella, Liverana Lauretti, Mario Rigante, Davide Mattavelli, Marco Ferrari, Jacopo Galli, Alessandro Olivi, Michele di Domenico, Francesco Doglietto

**Affiliations:** 1 Department of Neurosurgery, Papa Giovanni XXIII Hospital, Bergamo, Italy; 2 Technology for Health PhD Program, University of Brescia, Brescia, Italy; 3 Neurosurgery, Department of Neurosciences, Università Cattolica del Sacro Cuore, Rome, Italy; 4 Neurosurgery, Fondazione Policlinico Universitario Agostino Gemelli IRCCS, Rome, Italy; 5 Department of Pathology, Spedali Civili of Brescia, University of Brescia, Brescia, Italy; 6 Department of Neurology, Spedali Civili of Brescia, University of Brescia, Brescia, Italy; 7 Division of Neurosurgery, Department of Medical and Surgical Specialties, Radiological Sciences, and Public Health, Spedali Civili of Brescia, University of Brescia, Brescia, Italy; 8 Department of ENT, Fondazione Policlinico Universitario Agostino Gemelli IRCCS, Rome, Italy; 9 Unit of Otorhinolaryngology, Head and Neck Surgery, Department of Surgical Specialties, Radiological Sciences and Public Health, University of Brescia, Brescia, Italy; 10 Section of Otorhinolaryngology-Head and Neck Surgery, Department of Neuroscience, University of Padova, Padova, Italy; 11 Unit of Otorhinolaryngology-Head and Neck Surgery, Padua University Hospital, Padova, Italy

**Keywords:** cavernous sinus, cranial nerve, endoscopic skull base, intraoperative neuromonitoring, skull base surgery

## Abstract

**Introduction:**

Intraoperative neurophysiological monitoring (IONM) of extraocular cranial nerves (EOCN) during skull base surgery has expanded to reduce post-operative neurological deficits. However, multiple techniques exist, and comparative evidence supporting their routine use in endoscopic endonasal approaches (EEA) remains limited. The aim of the study is to evaluate the reliability, signal quality, and safety of different IONM techniques for CN III and VI during EEA, integrating a scoping literature review with a single surgeon’s clinical experience.

**Methods:**

A scoping review identified studies reporting EOCN monitoring during EEA. In parallel, a retrospective analysis was conducted in patients undergoing extended EEA with multimodal IONM. Monitoring modalities included free-run electromyography (fEMG), triggered electromyography (tEMG), electrooculography (EOG), and corticobulbar motor evoked potentials (CbMEPs). Direct muscle recordings were obtained using hook-wire electrodes or modified 90°-bent subdermal needle electrodes, while indirect monitoring relied on EOG. Signal quality, reproducibility, feasibility, and procedure-related complications were assessed.

**Results:**

Eleven studies involving 476 patients were included in the scoping review. In the clinical series, 51 patients underwent multimodal IONM. Direct EMG recordings provided stable and specific signals, whereas EOG showed a high artefactual component, limiting reliable nerve identification. CbMEPs yielded clear and reproducible responses in selected cases. Modified 90° bent dual subdermal needle electrode demonstrated superior signal quality and low impedance compared with hook-wire electrodes, which were associated with a higher rate of minor complications.

**Discussion:**

Multimodal IONM combining CbMEPs, tEMG, and fEMG is feasible and effective during EEA. Modified 90° bent dual subdermal needle electrode appear to offer the most reliable and safe monitoring strategy for EOCN.

## 1 Introduction

During transcranial microsurgical procedures, intra-operative neurophysiological monitoring (IONM) has become a standard tool for preserving neurological function (1–3). By improving intraoperative decision-making, it has contributed to making neurosurgical and skull base procedures safer and more effective, thereby offering the best possible outcomes for patients (1–3).

Among the various neuroanatomical regions, the cavernous sinus (CS) represents one of the most complex surgical areas due to the presence of intricate and delicate neurovascular structures. These include the extraocular cranial nerves (EOCN) – cranial nerve (CNs) III, IV, and VI - which may be directly involved by pathological processes in this region or indirectly injured during surgical manipulation, resulting in potential neurological injury (4–6).

The development of minimally invasive techniques, together with the rapid expansion of endoscopic skull base surgery, has further emphasized the need for IONM to reduce the risk of post-operative deficits. Several methods for EOCN mapping have been described, including electrooculography (EOG), free-run electromyography (fEMG), and trigger electromyography (tEMG) (7–14). Among these, the use of tEMG appears to be the most promising method compared with fEMG; however, there is no conclusive evidence demonstrating a reduction in post-operative complications (9, 13, 14). This limitation likely reflects the scarcity of robust statistical analysis and the limited size of available datasets in the current literature (9, 13, 14).

Unlike in transcranial surgery, its application in endoscopic endonasal approaches (EEAs) remains sparsely reported in the literature, highlighting a significant gap not only in its clinical application but also in defining the most appropriate monitoring methodology (7–14). In particular, no consensus has yet been reached regarding which neurophysiological approach offers the optimal balance among technical feasibility, reliability, and anatomical specificity in this setting.

In this context, the present study provides a twofold contribution: a scoping review on IONM techniques used during EEA for CS lesions, highlighting the existing gaps in the current evidence base, and a retrospective analysis of patients with skull base tumors and tumor-like lesions involving the CS, treated by the same surgeon using EEA and different monitoring strategies. These included a direct approach, based on electrodes placed on the extraocular muscles, and an indirect approach, based on EOG to assess eyeball movements. Direct stimulation responses, spontaneous and manipulation-induced muscle activity, and corticobulbar motor evoked potentials (CbMEPs) were assessed with both techniques.

## 2 Materials and methods

### 2.1 Search methods - scoping review

Given the expected heterogeneity of the available literature and limited number of eligible studies, a scoping review was conducted in PubMed, Scopus, and Web of Science on September 9, 2025, in accordance with the Preferred Reporting Items for Systematic Reviews and Meta-Analyses for Scoping Review (PRISMA-ScR) (15) with the aim of mapping the existing evidence and identifying knowledge gaps. The search strategy combined the following keywords and terms:”intraoperative neurophysiolog*”, “cranial nerv*”, “skull base surgery”; “intraoperative monitoring”, needle electrode*, mapping, and endoscop* (**Supplementary Table 1**). A total of 4116 records were found and imported into the software Zotero (version 6.0.31, Vienna, VA, USA) (**Figure 1**). After duplicate removal, three authors independently screened titles and abstracts. Full-text articles were then assessed for eligibility based on the predefined inclusion criteria, and relevant data were extracted and summarized. In case of uncertainty, an expert in IONM (M.d.D.) and the senior author (F.D.) were consulted.

**Figure 1:**
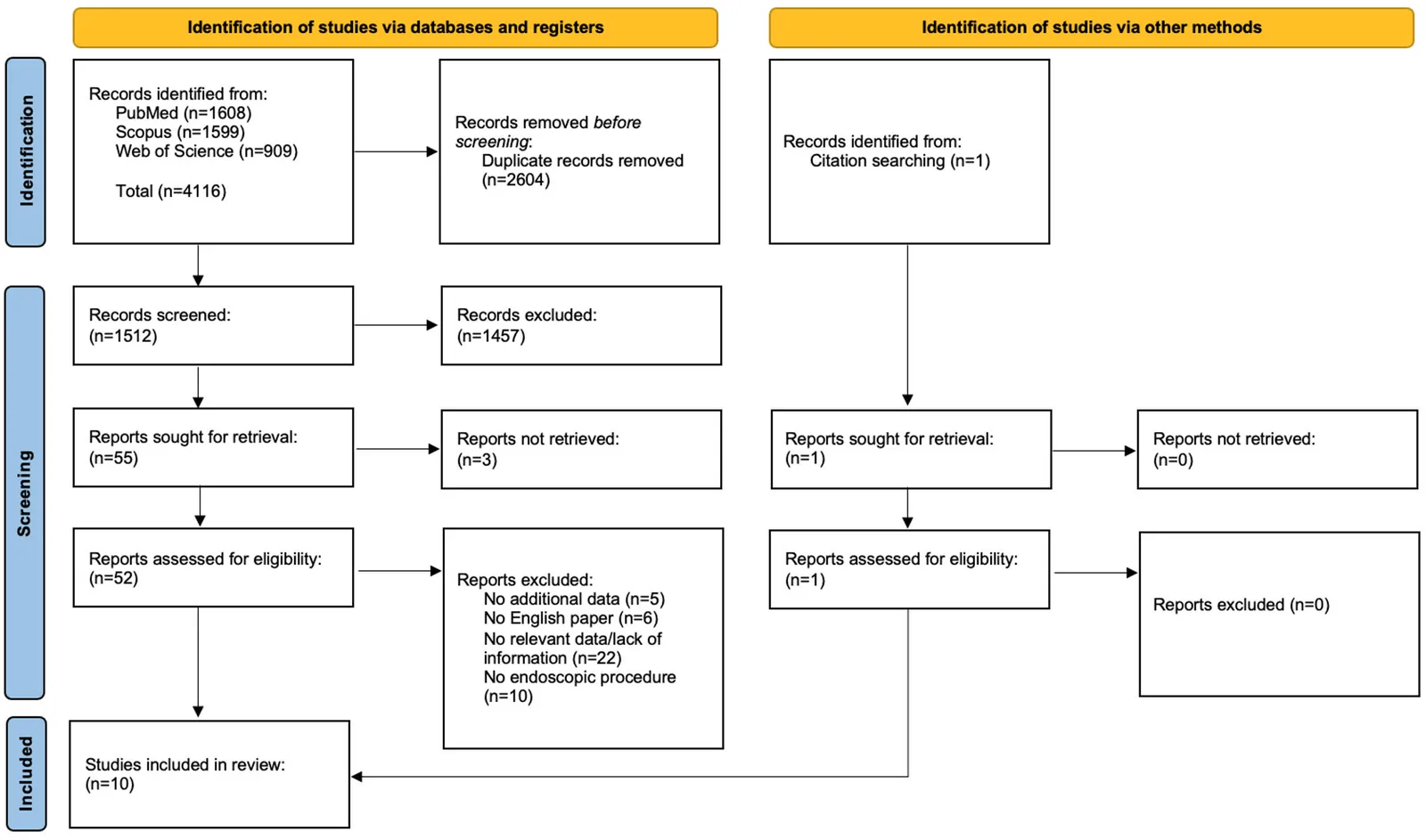
PRISMA flowchart.

### 2.2 Eligibility criteria and data extraction

The review focused on patients with skull base lesions undergoing EEAs, with IONM of CN III, CN VI, or CN IV. The analysis evaluated the monitoring techniques used, as well as their effectiveness and safety in clinical studies. Only clinical studies published in English were included.

Exclusion criteria were as follows: pediatric population (age <18 years), studies regarding IONM in microsurgical transcranial approaches, absence of EOCN monitoring during the procedure, lack of relevant clinical outcomes or safety data, and non-original articles, including reviews, meta-analyses, letters, conference papers, and preclinical studies on animals or cadavers.

For data extraction, the following information was analyzed: (1) authors and year of publication, (2) number of patients involved in each study and histology of the lesion (3) gender if specified, (4) type of endoscopic approach, (5) modalities of stimulation (EOG, fEMG, and tEMG), (6) recording electrode, (7) placement of the electrode, (8) type of stimulation (bipolar, monopolar), (9) nerves stimulated (sides), (10) stimulus intensity, (11) duration, (12) latency, (13) identification of the nerve, (14) CNs pre-operative deficits, (15) type of resection, (16) new post-operative deficits, (17) related complications (i.e., subconjunctival hemorrhage), and (18) follow-up.

Categorical variables were summarized as frequencies and percentages. Two-sided 95% confidence intervals for proportions were calculated using the Wilson score method because of the relatively small sample sizes. Analyses were performed using Jamovi version 2.3 (The Jamovi Project, version 2.3).

### 2.3 Clinical study

Fifty-one patients with complex skull base tumors and tumor-like lesions (including PitNETs, chordomas, chondrosarcomas, Rathke’s cleft cyst, and chondromyxoid fibroma) involving or extending to the parasellar region underwent EEA surgery with IONM performed by the same surgeon at the Department of Neurosurgery, Fondazione Policlinico Gemelli (Rome, Italy) and Spedali Civili of Brescia (Brescia, Italy) from 2017 to 2024. Informed consent for publication of clinical data and images was obtained from all patients, in accordance with the principles of the Declaration of Helsinki. The Institutional Review Board approved the study (Spedali Civili di Brescia, Brescia, Protocol Number 5071 – OMCPitNET; and Fondazione Policlinico Gemelli, Rome, Protocol Number 6546 - TYEPDD). For each patient, a retrospective analysis of medical records was performed. Inclusion criteria were: 1) patients with parasellar tumor extension at the potential risk of EOCN deficits due to the surgical approach, 2) aged > 18 years old, 2) complete information on clinic-radiological follow-up, 3) sufficient details regarding the IONM procedure, in order to compare the different techniques. Nineteen patients were excluded due to incomplete IONM data.

The anesthetic protocol included premedication with midazolam (0.2 mg/kg), induction using remifentanil (2–3 ng/ml in continuous infusion) and propofol (3-3.5 µg/ml), and maintenance with total intravenous anesthesia (TIVA) regimen consisting of propofol (2.5-3.0 μg/ml) and remifentanil (4–5 ng/ml). Muscle relaxants were administered only for endotracheal intubation (0.08 mg/kg vecuronium bromide) (16).

Intraoperative monitoring was performed using a 32-channel Medtronic NIM-Eclipse^®^ (Medtronic, Jacksonville, FL, USA) instrument. Extraocular muscles activation was studied using direct and indirect techniques. The direct method studies muscular activations using electrodes placed directly on the muscular belly, while the indirect one investigates muscular activation by studying eyeball movements. For the direct method, hook-wire electrodes (SGM™ Medical Monitoring, Spilt, Croatia) and different lengths of subdermal needles (Medtronic, Jacksonville, FL, USA and Inomed Medizintechnik, GmbH, Emmendingen, Germany) were used.

The hook-wire electrode consists of a flexible electrode guided by a hypodermic needle. After insertion of the electrode wire into the desired muscle, the hypodermic needle is withdrawn, leaving the flexible electrode’s wire securely fixed in the muscle. The connection cable is about 10 cm long, so that an extension cord is necessary (**Figure 2**).

**Figure 2:**
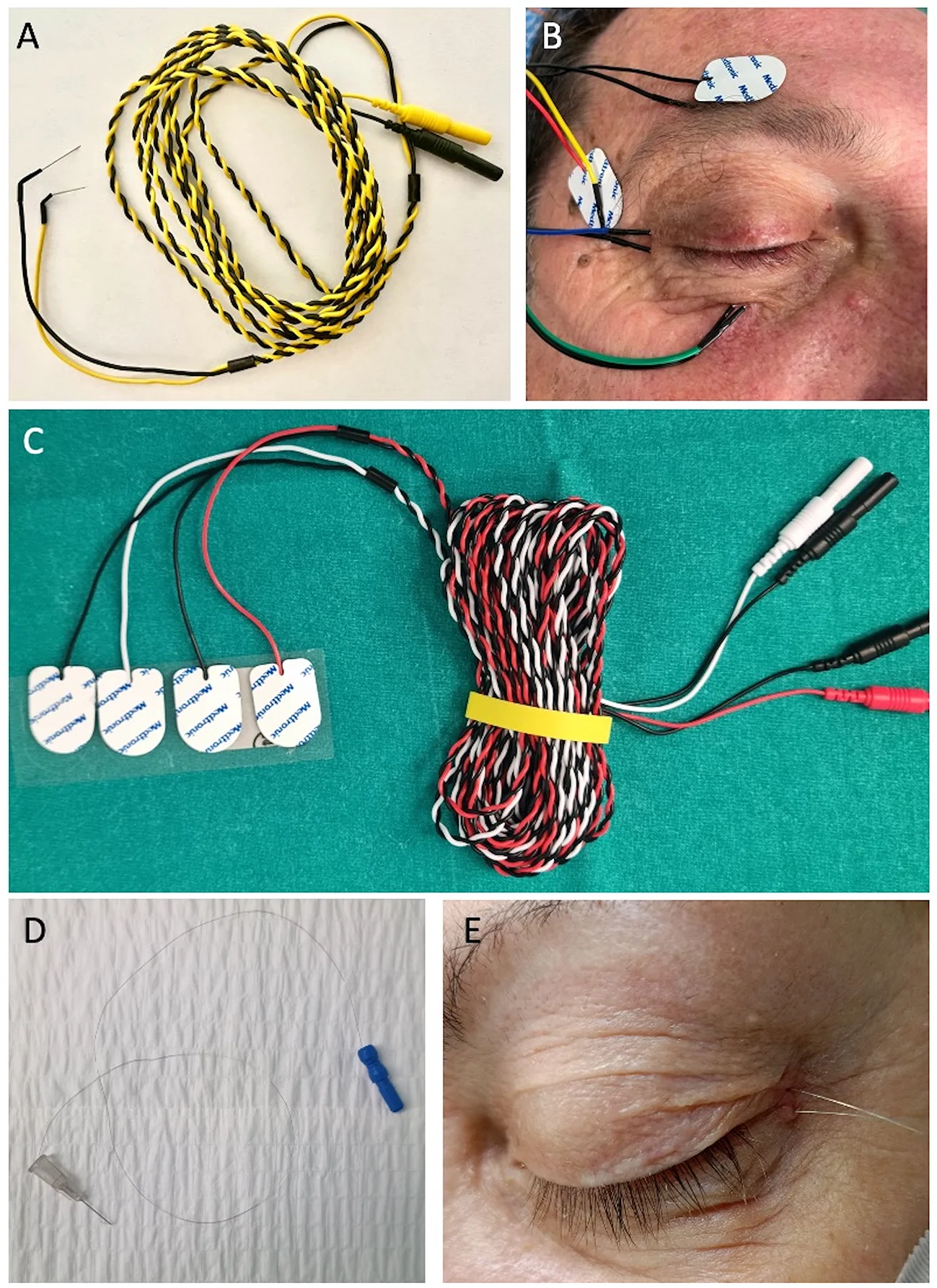
Needle for intraoperative monitoring and their positioning. **(A)** Image of the modified 90° bent dual subdermal needle electrode; **(B)** Placement of the needles on patient before endoscopic transnasal surgery; **(C)** Surface adhesive pad electrodes for EOG; **(D)** Hook-wire electrode composed of a flexible electrode guided by a hypodermic needle; **(E)** Placement of the hook-wire electrode.

A subdermal needle (27G, 13 mm and 15mm in length) was appropriately modified to improve stability, signal quality, and reproducibility during IONM of the extraocular motor pathways. The modified electrodes consisted of commercially available subdermal needles adapted for this purpose by creating a 90-degree bend at the exposed distal segment of the needle. This modification allowed more secure fixation, reduced displacement during draping and surgical manipulation, and facilitated a more favorable angle of entry into the muscle belly. (**Figure 2**).

Impedance was checked after placement and before stimulation. Optimal recordings were typically associated with impedance values below 1 kΩ, which correlated with higher signal-to-noise ratio and more reliable response identification. Conversely, elevated or unstable impedance suggested poor contact, malposition, partial dislodgement, or insufficient intramuscular penetration. In such cases, the electrode was repositioned, the insertion angle was corrected, or the needle was replaced if necessary.

Indirect monitoring (EOG) was performed by placing two active electrodes per eye, one positioned at the lateral canthus for the horizontal movements, and the other in the lateral part of the arcus superciliaris for vertical movements. The reference electrode was placed at the nasion. For 3 patients, EOG was performed using two different type of electrodes at the same time: surface adhesive pad electrodes (15x20 mm) (Medtronic, Jacksonville, FL, USA), and subdermal needle (Medtronic, Jacksonville, FL, USA) (**Figure 2**).

For direct monitoring electrodes, placement was performed using a transpalpebral freehand technique (or in case of doubt with the support of the neuronavigation system) before the surgical procedure under general anesthesia. After palpation of the bony rim of the orbital cavity, one (monopolar registration) or two (bipolar registration) electrodes were inserted in the superior, lateral, and inferior rectus. In monopolar recordings, a subdermal needle placed at the nasion was used as a reference. In direct IONM, only one of the described electrode types was used for each patient.

Stimulation (tEMG) was performed using a monopolar probe (Medtronic stimulator probe, Medtronic Xomed, Inc. Jacksonville, FL, USA). Single pulse, negative square waves, 500 µs pulse width, current intensities ranging from 0.1–10 mA were delivered. Higher stimulation intensities were used for intratumoral stimulation, allowing deeper current penetration and facilitating identification of neural structures located beneath the tumor. The analysis period lasted at least 30 ms, with sensitivity adjusted in relation to the response amplitude.

CbMEPs were performed to monitor extraocular motor pathways. Stimulating electrodes were corkscrew electrodes placed on the scalp in C3–Cz and C4–Cz (international 10–20 system) for right and left muscles respectively, using the double train technique to avoid false negatives, as specified in our previous publication (17).

Practical troubleshooting included verifying that the needle tip remained within the intended muscle belly after draping, minimizing cable traction, and securing the external portion of the electrode to prevent microdisplacement. If the recorded response was low amplitude, noisy, or nonreproducible, the first steps were to reassess depth, orientation, and impedance, and then to compare the trace with contralateral or alternative electrode recordings when available. In bilateral monitoring, symmetry of responses was also used as a practical internal control.

The following neurophysiological parameters were evaluated: signal quality, reliability of responses, and signal stability. Signal quality was defined by the signal-to-noise ratio; a “good-quality” signal displayed a low level of artifacts, allowing clear identification of neurophysiological activity. Signal stability was defined as the absence of significant changes in recordings throughout the observation period; in this study, a significant change was defined as an increase in latency of at least 10% or a decrease in amplitude of at least 50%. A reliable response (reproducibility) was defined as a signal that could be reproduced in at least two acquisitions (18).

For each patient, the following information was extracted: 1) demographic characteristics (age, and gender), 2) clinical history (including prior treatments), 3) imaging findings (including modified Knosp grade), 4) preoperative EOCN deficits and symptoms, 5) histopathological diagnosis, 6) type of IONM and needle employed, 7) surgical resection, 8) significant changes in IONM signals, and 9) post-operative complications.

Postoperative cranial nerve deficits were defined as any new or worsened dysfunction of CN III, IV, or VI identified after surgery and confirmed by clinical and neuro-ophthalmological examination. Outcome assessment included standardized postoperative neurological evaluation together with ophthalmological assessment of visual acuity, ocular motility, diplopia, ptosis, pupillary responses, and visual fields, with additional specialist review and neuroimaging performed when clinically indicated. Follow-up evaluations were conducted at serial outpatient visits and, when necessary, by additional clinical assessment. Deficits were considered transient when they resolved during follow-up and persistent when still present at the last available evaluation.

## 3 Results

### 3.1 Summary of scoping review

The scoping review included 11 articles (8–11, 14, 19–24) published from 2008 to 2023, describing 476 patients (**Figure 1**). Most cases were represented by PitNETs (n=199; 95% CI 37.5-46.3), followed by chordomas (n=73; 95% CI 12.3–18.8) and meningiomas (n=56). Among the various approaches described, the endoscopic transclival approach was the most frequently performed (45.5%, 106/233; 95% CI 39.2–51.9). The CS approach was described in 63.6% of articles (7/11; 95% CI 35.4–84.8), with 182 CS procedures performed (9, 11, 14, 19, 20, 23, 24). In 6 studies (54.5%; (95% CI 28.0–78.7), EMG was the most commonly used IONM technique, while EOG was employed less frequently, in 3 out of 11 studies (27.3%; 95% CI 9.7–56.6) and in two articles EOG and EMG were used together (7, 20). The stimulation intensity ranged from 0.1 to 16 mA, and in two studies it was measured in volts, with a range of 0.1 to 4 V. The duration of stimulation ranged from 100 to 500 microseconds.

The pre-operative neurological status was detailed in 8 articles (95% CI 43.4-90.3) (8–11, 19, 20, 22, 24). Specifically, deficits of the CN III were reported in 13 patients, CN IV in 2, and CN VI in 15 (11.2%). New post-operative deficits were observed in 60 patients (16.2%) (8, 9, 14, 19, 20, 24). In 32 cases, regression or resolution of the deficit was described at follow-up (8, 14, 19, 20, 24). An improvement of pre-operative deficit was described in 15 patients (9, 11, 19, 20). The results of surgical treatment, including the type of resection (gross total, subtotal, or partial), were reported on post-operative CT and/or MRI in 36.4% of the studies (4/11; 95% CI 15.2-64.6) (9, 19, 20, 22).

In 27.2% of articles (3/11), the occurrence of complications or adverse events associated with invasive needle electrode monitoring was investigated (10, 21). The complications reported included conjunctival hemorrhage, periorbital ecchymosis, corneal abrasion, ptosis, and upper eyelid edema (5.7%, 4/70; 95% CI 2.2-13.8). All complications were resolved at follow-up. A summary of the characteristics of the eleven papers included is described in **Supplementary Table 2**.

### 3.2 Clinical study

Of the 51 patients enrolled in the study, 19 were excluded due to incomplete monitoring data. Therefore, a total of 32 patients met the inclusion criteria (**Table 1**).

**Table 1:** Summary of the clinical features of patients affected by cavernous sinus lesions enrolled in the clinical study.

N° of Patients	32
Median age at the surgery	52.9 (20–79 yo)
Male	20 (62.5%)
Female	12 (37.5%)
Pre-op Deficits	14 patients (43.8%)
CN III Palsy	7
CN V1 Palsy	2
CN VI Palsy	9
Visual Impairment	11
Headache	10
Tumor Histology	
PitNETs	24 (75%)
Chordomas	6 (18.8%)
Rathke’s cleft cyst	1 (3.1%)
Chondromyxoid fibroma	1 (3.1%)
CNs monitored	98
CN III	15 bilaterally, 17 monolaterally
CN IV	2 bilaterally*
CN VI	15 bilaterally, 13 monolaterally
Range stimulus intensity	0.1–10 mA
Types of stimulations	
EOG	4
monopolar-registered EMG signal	7
bipolar-registered EMG signal	6
90°-bent bipolar-registered EMG signal and CbMEP	12
90°-bent bipolar-registered EMG signal, EOG, and CbMEPs	3
Type of Resection	
GTR	13
STR	17
Cyst Fenestration	1
Complications	
Transient worsening of pre-operative deficit	2 (1 invasive bipolar curved needle, 1 Hook-wire electrode)
Subconjunctival hemorrhage	2 (invasive bipolar curved needle)
Periorbital ecchymosis	1 (invasive bipolar curved needle)
New cranial nerve deficits	4 (1 invasive bipolar curved needle, 3 Hook-wire electrode)

CN, Cranial Nerve; GTR, Gross Total Resection; STR, Subtotal Resection; *, monopolar-registered EMG signal with Hook wire needle.

The cohort comprised 20 men (62.5%) and 12 women (37.5%), with a median age of 52.9 years (range, 20–79) and a follow-up ranging from 3 to 39 months. Seventeen patients (53.1%) had previously undergone surgery and/or non-surgical therapy. Preoperative CN deficits were present in 14 cases: CN III palsy in 7 patients (5 partial and 2 complete), CN VI palsy in 9 patients (bilateral in one), and hypoesthesia in the V1 territory in 2 patients.

Additional preoperative symptoms included visual impairment (n=11; 34.4%) and headache (n=10; 31.3%). Less common findings included acromegalic facial features (n=1), cushingoid appearance (n=1), decreased libido (n=2), memory disturbances (n=1), and gait disturbance (n=1). Most lesions were pituitary neuroendocrine tumors (PitNETs, n=24; 75%), followed by chordomas (n=6; 18.8%), Rathke’s cleft cyst (n=1), and chondromyxoid fibroma (n=1).

A total of 98 cranial nerves were monitored: CN III bilaterally in 16 patients (n=32 nerves) and unilaterally in 17; CN IV bilaterally in 2 patients; and CN VI bilaterally in 16 and unilaterally in 13. Stimulation intensity ranged from 0.1 to 10 mA. Hook-wire electrodes were used for monopolar EMG recordings (fEMG and tEMG) in 5 patients and bipolar EMG in 5. Modified 90°-bent subdermal needles were used in 15 patients for EMG, tEMG, and CbMEP simultaneously. Long curved needle electrodes were used in 5 cases for EMG, tEMG, and CbMEP signals. Both modified 90°-bent electrodes and long curved needles were always used for bipolar EMG recording. Using hook-wire electrodes, tEMG produced positive responses in 6 of 10 cases, though the stimulated nerve could not always be clearly identified. In 4 cases, stimulation was not feasible because of the very low quality of the signal acquired, and in these cases impedance values ranged from 7.2 kΩ to >50 kΩ. With the 90°-bent subdermal needles, clear and reproducible muscular activations were recorded in all but 2 cases, in which needle malposition or displacement occurred during draping. These electrodes, except for the cases mentioned above, always showed impedance values lower than 1 kΩ, which helped to maximize the signal-to-noise ratio, allowing for clear and distinguishable responses. The long-recurved needle electrodes produced selective muscular activation in 4 cases; in one, no response was observed.

Out of 10 patients who underwent EOG, complete data was available for 4 patients (14 nerves). Compared with EMG, EOG signals showed high artifact levels and poor reproducibility, even in the absence of stimulation. EMG signals, by contrast, were stable and specific. No significant differences in signal quality were observed between the 13-mm and 15-mm curved needles.

CbMEPs recorded with 90°-folded bipolar electrodes yielded excellent, consistent responses in 11 patients. Signal quality was unstable and poorly interpretable in 3 patients affected by preoperative CN deficits (1 patient with left CN VI deficit, 1 patient with left CN III deficit, and 1 patient with both right CN III and right CN deficits). In contrast, EOG-based CbMEP monitoring was unreliable due to excessive artifacts, except for vertical eye movements in one case (**Figure 3**; **Supplementary Tables 3**, **4**).

**Figure 3:**
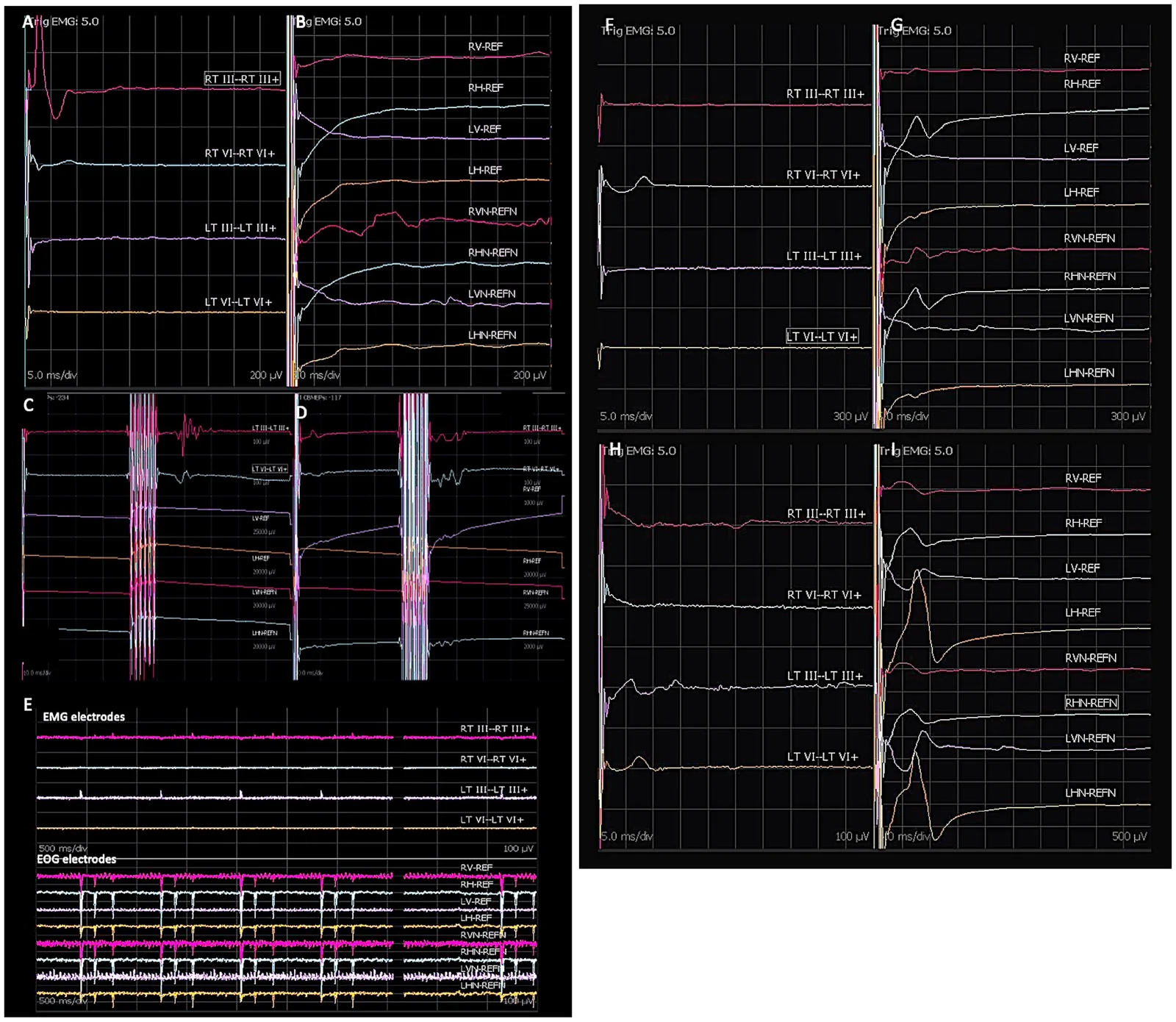
Intraoperative monitoring signal variations. **(A)** Example of right CN III direct stimulation recorded by EMG channels with a clear muscular activation. **(B)** Example of right CN III direct stimulation recorded by EOG without a clearly recognizable response. C-D. Acquisition of CbMEPs using different techniques. **(C)** a clear and stable response from EMG electrodes, **(D)** The artefactual component in EOG require amplification about 20–25 mV that make it impossible to recognize clear responses except for (in this case) for R VN-REFN. **(E)** Raw EMG signals recorded from all EMG electrodes compared with EOG electrodes (below). The artefactual component recorded from EOG channels without any surgical stimulus applied is evident. **(F)** Example of right CN VI activation recorded by EMG channels that show a selective response in the RT VI channel. **(G)** Example of right CN VI activation recorded by EOG channels: a diffused response is observed. A clear nerve identification was not possible. **(H)** Example of left CN VI activation recorded by EMG channels with an activation of both left CN III and CN VI; **(I)** Example of left CN VI activation recorded by EOG channels with a diffused activation with a wider amplitude on left horizontal channels (both adhesive and needle electrodes). The nerve was recognized using stimulation and anatomical observation. H-REF, Horizontal Movements; LT, Left; RT, Right; V-REF, Vertical Movements; III, Oculomotor nerve; VI, abducent nerve.

Gross total resection (GTR) was achieved in 13 patients, subtotal resection (STR) in 17, and cyst fenestration in one. Regarding complications, 6 cases of cranial nerve deficit (18.8%, three of which were transient) (**Table 2**) and 3 (9.4%) complications associated with IONM (subconjunctival hemorrhage and periorbital ecchymosis) were reported.

**Table 2:** Summary of the 6 patients with post-operative cranial deficits according to type of lesion, type of intraoperative monitoring and significant variation of signal.

Case N°	Histological diagnosis	Modified knosp	Prior surgical treatment	Pre-operative CN deficits	Type of IONM	Type of needle	Significant intraoperative changes in IONM signals	Post-operative CN deficits
Case 1	Pit-NET	2 (*)	Y	N	EMG, tEMG, CbMEP	90°-bent bipolar electrodes	N	tCN VI
Case 2	Pit-NET	4 (Ls)	Y	Y – Bilateral partial CN III	EMG, tEMG, CbMEP, EOG	90°-bent bipolar electrodes, and Surface electrodes	irritation of inferior rectus muscles	pCN III
Case 3	Pit-NET	4 (Ls)	Y	N	fEMG III	Hook-wires	Signs of Irritation during diving technique and exploration of surgical cavity	pCN III
Case 4	Pit-NET	4 (Rs)	Y	Y – right CN III	EMG III, IV	Hook-wires	Signs of Irritation during hemostasis	pCN III
Case 5	Pit-NET	4 (Ls)	Y	N	EMG III, IV	Hook-wires	Spontaneous irritation of inferior rectus muscles	tCN III
Case 6	Pit-NET	3B (Ls)	Y	N	fEMG	Hook-wires	N	tCN III

CN, Cranial Nerve; fEMG, free run Electromyography, IONM, Intra-Operative Neurophysiological Monitoring; Ls, Left Side, Pit-NET, Pituitary Neuro Endocrine Tumor; Y, Yes; N, No; p, permanent; Rs, Right side; t, transient; tEMG, triggered Electromyograph; *, both sides.

Among those undergoing resection using invasive bipolar curved needle monitoring, one experienced deterioration of a pre-existing CN III palsy, presenting as impaired upward gaze, and another developed a new CN VI palsy without intraoperative signal changes, which resolved completely within 2 months. Minor complications included subconjunctival hemorrhage in 2 patients and periorbital ecchymosis in one, all of which resolved within one month (**Figure 4**).

**Figure 4:**
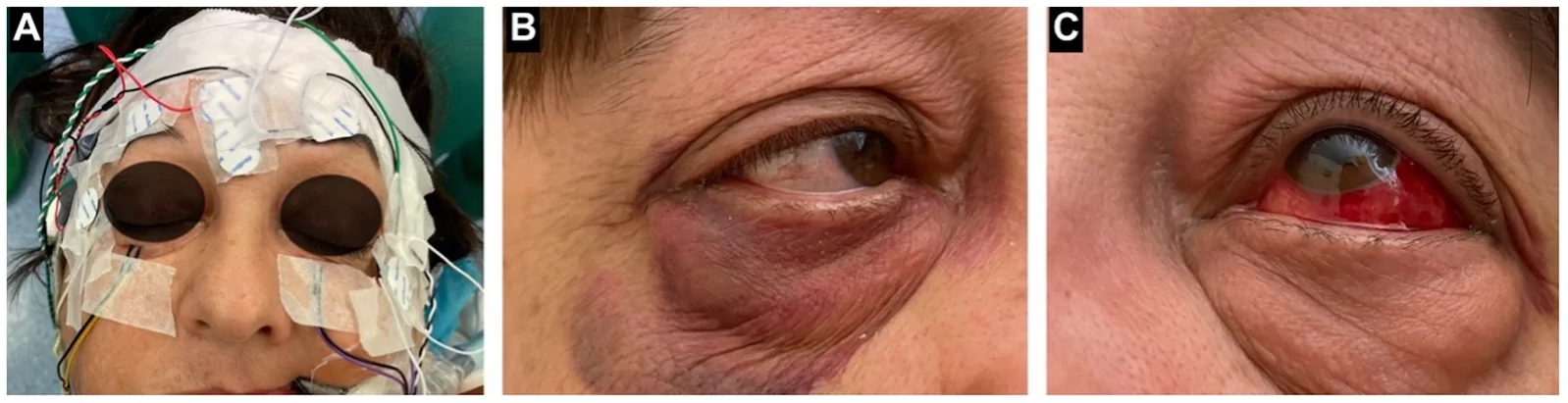
Intraoperative setting and post-operative complications. **(A)** Preoperative positioning of the needles and surface adhesive pad electrodes before an EEA surgery. **(B)** Periorbital ecchymosis after EEA due to the positioning of the modified 90° bent dual subdermal needle electrode. **(C)** Subconjunctival hematoma that completely resolved without sequelae after 30 days from the surgery.

Hook-wire electrode–related events, which were higher but statistically nonsignificant, included palpebral ptosis (n=1), transient diplopia (n=2), and worsening of a pre-existing upward gaze deficit associated with rectus muscle hypofunction (n=1). One patient died from systemic complications during post-operative rehabilitation. Tumor regrowth occurred in 59% of patients with STR, requiring adjuvant treatment. Six received radiotherapy (proton beam or gamma knife), one of whom underwent repeat transcranial resection. Two patients underwent adjuvant chemotherapy, with one requiring reoperation after 2 years, and another patient underwent repeat procedure at 3 years after the initial surgery.

## 4 Discussion

This study combines a scoping review with institutional experience to refine current IONM strategies and identify a reliable, reproducible approach for EOCN assessment. In our series, we used modified dual subdermal 90-degree bent needles of two lengths (13 mm and 15 mm) together with CbMEPs to monitor the extraocular pathways during endoscopic transnasal surgery of the CS region.

IONM during EEA represents a relatively underexplored aspect of skull base surgery. In recent decades, the growing use of endoscopic approaches has been driven by their minimally invasive nature, improved visualization, and broad panoramic exposure (25, 26), particularly for tumors with parasellar or cavernous sinus extension. In this setting, preserving cranial nerve function while achieving maximal safe resection remains a major surgical challenge, and IONM may contribute meaningfully to improved functional outcomes (7, 27).

However, despite its increasing clinical adoption, the available evidence remains limited and methodologically heterogeneous. Our scoping review (8–11, 14, 19–24) showed that most published studies consist of small retrospective case series with substantial variability in tumor pathology, surgical approaches, monitoring techniques, stimulation parameters, outcome definitions, and follow-up duration. This heterogeneity precludes meaningful quantitative comparison between studies and limits the generalizability of the available evidence. Furthermore, standardized reporting of neurophysiological parameters and postoperative cranial nerve outcomes was frequently lacking, making direct comparison across studies difficult and underscoring the need for more standardized methodological and reporting practices in future investigations.

### 4.1 Electrode type

Electrode choice is a critical component of IONM. Surface electrodes, although non-invasive and suitable for EOG, are limited by higher impedance at the skin–electrode interface, which may reduce signal fidelity (4, 8, 28–30). In contrast, needle electrodes provide more selective recordings and a better signal-to-noise ratio, enabling more reliable detection of the low-amplitude electrical activity generated by the extraocular muscles (4, 8, 28–31). For these reasons, needle electrodes remain the preferred option, despite the technical difficulty of placement in small, deep, and anatomically complex muscles (32). Moreover, it may also cause iatrogenic effects, including hematoma, and rarely critical complications such as globe injury or perforation, and electrode displacement (10, 19, 21, 23).

Most published series report the use of 10–13 mm subdermal needles electrodes (14, 21, 24),, and 7-mm superficial electrodes have been proposed as a less invasive alternative (21), though at the cost of more false positives and missed tEMG responses, limiting their reliability (21). It should be noted that 13–15 mm subdermal needles may not reliably reach all extraocular muscles in every patient (33–35). This has significant implications for the interpretation of recorded signals, which likely reflect a combination of muscular and perimuscular activation rather than a uniform sampling of the target muscle.

Conversely, longer electrodes (e.g., 25 mm) (36, 37) have been shown to provide larger amplitudes and better discrimination than standard 13-mm electrodes when deeper muscles can be reached, without a relevant increase in complications, supporting the use of longer needles in selected cases.

Hook-wire electrodes carry an additional drawback: the combination of a very thin shielding layer and the need for an extension cable can result in elevated and unstable impedance, increasing the signal-to-noise ratio and making consistent, clearly identifiable muscle responses difficult to obtain. In our experience, most neurological complications occurred in association with the use of these thin needles, suggesting that their unreliable impedance may compromise monitoring and increase the risk of unrecognized nerve injury.

Our findings align with these observations. The modified 15-mm and 13-mm twisted dual subdermal needles consistently yielded clear muscular activation with impedance below 1 kΩ, with no qualitative difference in signal morphology between the two lengths. The longer needles reached the muscle belly more effectively albeit associated with a small number of positioning-related complications that resolved spontaneously (2 cases of subconjunctival hemorrhage and edema with periorbital ecchymosis resolved in 30 days). In contrast, superficial adhesive electrodes and hooked needles produced uninterpretable signals in all cases and were associated with post-operative CN deficits (4 cases), underscoring the limitations of overly superficial or unstable recording techniques when reliable intraoperative feedback is required.

### 4.2 Types of monitoring techniques

EMG was first introduced for intraoperative assessment of EOCN functions during CS surgery in the 1980s (38), and its use has progressively expanded. Both fEMG and tEMG were used in a majority of studies (10, 21, 22, 24).

fEMG provides real-time warning of CN irritation (14) through spontaneous neurotonic discharge without the need for external stimulation, thereby guiding the surgeon during surgical manipulation (7). However, its predictive value remains controversial (14, 36, 39) because of susceptibility to noise and artifacts, and because significant structural injury (including transection) may occur without conspicuous fEMG changes (38). This is consistent with our observation of new post-operative deficits despite the absence of major intraoperative fEMG changes, as demonstrated in our study, reinforcing that fEMG should not be relied on as a standalone monitoring tool.

tEMG provides complementary value by mapping nerve trajectories and assessing functional integrity, and its combination with fEMG may improve intraoperative decision-making and reduce the risk of injury, even though its impact on long-term outcomes is not uniformly demonstrated (9, 10, 22, 40).

Although EOG offers a simpler and less invasive alternative (8, 41) because it relies on surface recordings, it is less sensitive and less specific than EMG and often fails to localize the source of the signal precisely (30).

In our simultaneous EOG and EMG recordings, EOG produced diffuse activation that was not as clearly interpretable as the corresponding tEMG signals, which supports the view that EOG is better suited as an adjunct than as a replacement for EMG. In addition, while EMG has been extensively researched and is widely regarded as the gold standard for monitoring extraocular motor nerves, EOG has not received the same level of attention or validation. This lack of data contributes to uncertainty regarding its reliability and efficacy in surgical settings, further cementing EMG as the preferred method.

Last but not least, the complementary use of CbMEPs with other monitoring modalities is increasingly supported. Recent data (42) on transcranial middle skull base procedures indicate that combining direct nerve stimulation with CbMEPs yields reproducible baselines in ~50% of cases and high diagnostic performance for predicting post-operative palsy (for CN III, CbMEP sensitivity 66.7% and specificity 100%; for CN VI, sensitivity 75.0% and specificity 96.8%). These results are consistent with our preliminary findings and conceptual framework, showing that CbMEPs yield monitoring data for the EOCN that closely correlate with post-operative deficit severity (Spearman ρ = 0.94 for CN III and ρ = 0.88 for CN VI) (42). This supports the view that CbMEPs, when combined with other intraoperative techniques, provide highly specific feedback that is becoming increasingly essential for best clinical practice in skull base surgery.

Beyond design new electrode variants, future developments in IONM are likely to focus on improving signal interpretation, acquisition, and clinical decision support (43). Thanks to the introduction of artificial intelligence in the clinical practice, alternative approaches are emerging such as advanced digital signal processing, automatic artifact rejection, machine-learning-assisted classification, and fusing multimodal data could improve the reliability of intraoperative recordings, facilitating more informed surgical decision-making (43). Although these technologies are still under investigation, they could represent promising directions for future clinical research.

## 5 Limitations

Given the retrospective design, limited sample size within individual monitoring subgroups, and the heterogeneity of the pathologies treated, formal comparative statistical analyses between monitoring techniques and electrode types could not be performed. Therefore, our findings should be interpreted as descriptive and hypothesis-generating rather than as evidence from a direct comparative analysis between monitoring techniques. Nonetheless, the descriptive evaluation of different monitoring approaches within the same surgical setting provides practical insights into their respective technical advantages and limitations.

The study reflects a single-surgeon series, which may reduce inter-operator variability but also limits generalizability, since technical preferences, operator expertise, electrode placement strategy, and intraoperative interpretation of neurophysiological signals may not be reproducible across other surgeons or institutions. Additionally, 19 of the 51 initially identified patients were excluded due to incomplete monitoring data. Although this was necessary to ensure reliable extraction of intraoperative neurophysiological variables, it may have introduced selection bias, as the final cohort could differ systematically from the initially eligible population in terms of tumor complexity, operative difficulty, or monitoring quality. This may have affected the representativeness of the analyzed cohort and therefore reduced the external validity and generalizability of the findings. The limited number of EOG cases and absence of prospective, randomized comparisons also preclude definitive statements regarding the superiority of one monitoring strategy over another, underscoring the need for larger, multicenter prospective studies.

## 6 Conclusion

The use of a modified 90° bent dual subdermal needle electrode, combined with simultaneous neurophysiological techniques (CbMEPs, fEMG, and tEMG), was associated with favorable intraoperative signal quality and a low rate of postoperative EOCN complications in our series. However, given the limited sample size, retrospective design, absence of controlled comparisons, patient heterogeneity, and lack of formal statistical power, these findings should be interpreted as descriptive rather than as evidence from a direct comparative analysis, and no definitive claim of superiority over alternative techniques can be made.

Together with the existing literature, our results support the potential role of multimodal CN monitoring during EEA and encourage further investigation aimed at integrating standardized IONM protocols into endoscopic skull base practice to improve intraoperative decision-making and support preservation of CN function. Future prospective multicenter studies involving larger patient cohorts are warranted to adopt homogeneous neurophysiological outcome measures and ensure uniform reporting of postoperative cranial nerve function. Such efforts would facilitate comparisons across institutions, improve the quality of available evidence, and provide stronger evidence regarding the clinical effectiveness of different intraoperative monitoring strategies.

## Data Availability

The raw data supporting the conclusions of this article will be made available by the authors, without undue reservation.

## Supplementary material

Supplementary Table 1 Complete search strategy used for the scoping review.

Supplementary Table 2 Summary of the studies included in the scoping review. Abbreviations: Bipol, Bipolar; Chond., Chondrosarcoma; Chol. Gran., Cholesterol Granuloma; Chord., Chordomas; CS, Cavernous Sinus; F, Female; f-EMG, free-run Electromyography; GTR, Gross Total Resection; IR, Inferior Rectus; l, left; LR, Lateral Rectus; M, Male; Men. Meningioma; Mono, Monopolar; MR, Medial Rectus; PitNET, Pituitary NeuroEndocrine Tumor; PR, Partial Resection; r, Right, SOM, Superior Oblique Muscle; SR, Superior Rectus; V, Volt; tEMG, trigger electromyography; ^ç^, Number and side of nerve monitoring not specified; * the type of IONM is deducted from the description of the article.

Supplementary Table 3 A summary of the advantages, disadvantages, and complications for each type of IONM electrode.

Supplementary Table 4 A summary comparing the signal characteristics for each different electrode types.
