# Correction: Neurophysiological monitoring of cranial nerves III and VI in endoscopic skull base surgery: scoping review and comparative study

**DOI:** 10.3389/fonc.2026.2012726

**Published:** 2026-10-13

**Authors:** Simona Serioli, Ginevra D’Onofrio, Federico Valeri, Alberto Pietrantoni, Laura Broglio, Ludovico Agostini, Pier Paolo Mattogno, Vito Stifano, Marco Maria Fontanella, Liverana Lauretti, Mario Rigante, Davide Mattavelli, Marco Ferrari, Jacopo Galli, Alessandro Olivi, Michele di Domenico, Francesco Doglietto

**Affiliations:** 1 Department of Neurosurgery, Papa Giovanni XXIII Hospital, Bergamo, Italy; 2 Technology for Health PhD Program, University of Brescia, Brescia, Italy; 3 Neurosurgery, Department of Neurosciences, Università Cattolica del Sacro Cuore, Rome, Italy; 4 Neurosurgery, Fondazione Policlinico Universitario Agostino Gemelli IRCCS, Rome, Italy; 5 Department of Pathology, Spedali Civili of Brescia, University of Brescia, Brescia, Italy; 6 Department of Neurology, Spedali Civili of Brescia, University of Brescia, Brescia, Italy; 7 Division of Neurosurgery, Department of Medical and Surgical Specialties, Radiological Sciences, and Public Health, Spedali Civili of Brescia, University of Brescia, Brescia, Italy; 8 Department of ENT, Fondazione Policlinico Universitario Agostino Gemelli IRCCS, Rome, Italy; 9 Unit of Otorhinolaryngology, Head and Neck Surgery, Department of Surgical Specialties, Radiological Sciences and Public Health, University of Brescia, Brescia, Italy; 10 Section of Otorhinolaryngology-Head and Neck Surgery, Department of Neuroscience, University of Padova, Padova, Italy; 11 Unit of Otorhinolaryngology-Head and Neck Surgery, Padua University Hospital, Padova, Italy

**Keywords:** cranial nerve, intraoperative neuromonitoring, skull base surgery, cavernous sinus, endoscopic skull base

In the **Acknowledgments**, the statement “Ministero della Salute – Ricerca Corrente 2026” was erroneously omitted. The original version of this article has been updated.

