# Supplementary material for: Neurophysiological monitoring of cranial nerves III and VI in endoscopic skull base surgery: scoping review and comparative study
Source: Front Oncol. 2026 Sep 14;16:1831926. doi: 10.3389/fonc.2026.1831926 (PMC13616701; doi:10.3389/fonc.2026.1831926)
Supplement: Supplementary file 1 [file Table1.docx]

Supplementary Material

**Supplementary Table 1**

**Keywords**

Intraoperative neurophysiolog* AND cranial nerv*

Intraoperative neurophysiolog* AND skull base surgery

Intraoperative monitoring AND cranial nerv*

Intraoperative monitoring AND skull base surgery

Intraoperative neurophysiolog* mapping AND cranial nerv*

Intraoperative neurophysiolog* mapping AND skull base

Needle electrode* AND cranial nerv*

Intraoperative neurophysiolog* AND cranial nerv* AND endoscop*

Intraoperative neurophysiolog* AND skull base surgery AND endoscop*

Intraoperative monitoring AND cranial nerv* AND endoscop*

Intraoperative monitoring AND skull base surgery AND endoscop*

Intraoperative neurophysiolog* mapping AND cranial nerv* AND endoscop*

Needle electrode* AND cranial nerv* AND endoscop*

**Supplementary Table 1**

Complete search strategy used for the scoping review.
