# Supplementary material for: Neurophysiological monitoring of cranial nerves III and VI in endoscopic skull base surgery: scoping review and comparative study
Source: Front Oncol. 2026 Sep 14;16:1831926. doi: 10.3389/fonc.2026.1831926 (PMC13616701; doi:10.3389/fonc.2026.1831926)
Supplement: Supplementary file 2 [file Table2.docx]

Supplementary Material

2. Supplementary Tables

Table 2

| **First**  **Author, Year of Publication** | **Histology of the skull base tumor  (n° of procedures)** | **N° and gender (n, %)** | **Type of Approach (n, %)** | **Type of stimulation (EOG, tEMG, fEMG)** | **Types of Recording Electrode (dimensions)** | **Placement of electrode** | **Electrical Stimulation (i.e., bipolar, monopolar)** | **Nerve monitoring (sides)** | **Stimulus Intensity mA/V (range)** | **Durations (μs, range)** | **Latency**  **(ms)** | **Identification of the nerve (n, %)** | **Pre-op deficits (n, %)** | **Type of resection** | **New Post-op deficits (for each nerve) (n, %)** | **Related complications (n, %)** | **Follow up**  **(n for patient)** |
| --- | --- | --- | --- | --- | --- | --- | --- | --- | --- | --- | --- | --- | --- | --- | --- | --- | --- |
| Kitano et al (2008) [19] | 36 PitNETs  41 CS procedures | 36 (16M; 20F) | NS | t-EOG* | disc-shaped motion sensor | upper eyelid overlying the eyeball | mono | CN III, CN IV, CN VI  Number and side of nerve monitoring NS | NS | 500 | NS | NS | 8.33%  3 double vision (1 of 3 complete CN III palsy) | 33 GTR; 3 STR | 27%  11 extraocular palsy | 0 | Post-op. transient deficits (n=11) resolved within the 3 months  Pre-op deficits (n=3) improved |
| Kawamata et al (2013) [8] | 100 PitNETs | 100  (38M; 62F) | endonasal endoscope-assisted transsphenoidal microsurgery | EOG-EMG | Surface electrodes | upper eyelids (vertical traces), 1 in the middle of forehead reference, 1 in right cheek as ground; in 30 pt additional electrodes at the outer corners (horizontal records) |  |  |  |  |  |  | 0 | NS | 1%  1 patient CN VI palsy | NS | Regression |
| Thirumala et al (2013) [14] | 43 Men., 25 Chord.,  37 PitNETs,  6 Chond.  12 angiofibroma  77 others | 200  (88M; 112F) | 51 transellar,  91 transclival,  13 transplanum,  12 transcavernous,  12 transpterygoidal,  21 others. | f-EMG | intramuscular needle  (13mmx0.4) | CN III: MR  CN IV: SO  CN VI: LR | NA | 219 CN III (44l, 43r, 66 both)  192 CN IV  (38l, 40r, 57 both)  285 CN VI (48l, 41r, 98 both) | NA | NA | NA | NS | NS | NS | 3 CN III deficit  2 CN IV deficit  11 CN VI deficit | NS | Post-op deficit (1 CN IV, 2 CN VI) improved |
| San-juan et al (2014) [10] | 8 clival lesions (4 chord.; 2 meningiomas; 1 schw. 1 nasopharyngeal carcinoma) | 8 (2M; 6F) | 2 transsellar + transclival;  3 transclival;  2 transperygoid, 1 transclival+ transodontoid | t-EMG  f-EMG | intramuscular needle (electrodes sutured through the muscle) | CN VI:LR | mono | 16 CN VI (8l and 8r) | 0.1-4.0 mA | 500 | 1.5 (+-0.4) | 16 | 62 %  3 CN VI paresis  2 hemianopsia | NS | 0 | 1 subconjuntival hematoma | subconjuntival  hematoma resolved at 2 months FUP |
| Messerer et al (2016) [22] | 3 chordomas, 3 Men., 3 metastatic lesions, 1 chondroma, 1 chondrosarcoma.  3 CS procedures | 11 (4M- 7 F) | 8 EEEA;  3 EEEA+ transcranial | EOG (CN III+ CN VI) + t-EMG  f-EMG (CN IV) | Surface electrodes + intradermal needle | NS | mono | CN III, CN IV, CN VI ^ç^ | 1-5 mA | 200 |  | NS | 9%  1 Unilateral decreased visual acuity | 5 GTR, 4 STR, 2 PR | 0 | NS | NS |
| Shkarubo et al (2017) [9] | 6 chord., 3 Schw.  4 CS procedures | 9 (4M-5F) | 3 posterior extended transclival+ 5 extended lateral + 1 posterior extended transclival+extended lateral | t-EMG  f-EMG | intramuscular needle | CN III: SR, IR  CN VI: LR | Mono and bipol. | In 4 patients CN III,  in 7 patients CN VI ^§^ | 2-16 mA | 100 | 0.1 | 4 CN III; 7 CN VI | 88,8%  III (3 paresis; 1 plegia); IV (1 paresis); VI (3 paresis) | 6 GTR, 2 STR, 1 PR | 10%  1 patients  CN VI plegia | NS | Post-op deficit (n=1) improved  Pre-op deficits (n=2) improved |
| Sakata et al (2018) [23] | 1 PitNET  1 CS procedure | 1 M | NS | t-EOG* | Piezoeletric device (11 mm) | taped onto the patient’s eyelids | mono | 1  CN III (1r) | 1.5 mA | 250 | NS | NS | NS | NS | 0 | 0 (0%) | 0 (0%) |
| Shkarubo et al 2018 [20] | 11 chord.; 5 Schw.;  2 Men.; 3 PitNETs; 1 cholesteatoma; 1 chondroid chordoma)  7 CS procedures | 23 (7M; 16F) | 11 posterior extended transclival; 8 extended lateral; 3 transsellar + extended transclival; 1posterior extended transclival + extended bilateral | t-EMG | Intramuscolar needle | NA | Bipol. | CN III (7l, 5r)  CN VI (12l, 9r) | 2-16 mA | 100 | 0.1 | CN III 12; CN VI 23 | 65,2%  5 CN III (4 paresis 1 plegia) ;1 CN IV (paresis); 9 CN VI (paresis) | 18 GTR; 4 STR | 21.7%  5 patients:  1 CN III (increased paresis)  4 CN VI (increased paresis) | NS | Post-op deficit (n=2 – CN VI paresis) regressed  Pre-op deficits (n=9, 3 CN III, 6 CN VI) improved |
| Jeong et al (2021) [11] | 2 PitNETs, 2 Schw., 1 Craniop., 1 hemangiopericytoma, 1 angioleiomioma | 7  (2M - 16F) | NS | t-EOG | Surface electrodes | upper and lower eyelids (vertical traces), lateral and medial eye corner (horizontal traces) | Bipol. | 8  5 CN III;  3 CN VI. | 0.1-5 mA | 200 | 0.4–0.5 | NS | 14,2%  1 CN III palsy | NS | 0 | NS | improved |
| Adams et al (2023) [21] | NA ("skull base tumors") | 25 | NS | t-EMG  f-EMG | intramuscular needle (13mm x 0.4mm) and shorter (7mm x 0.4mm) | CN III: MR, medial canthus; CN IV: SO, supraorbital notch; CN VI: LR, lateral canthus | Mono | 71  19 CN III (6l, 13r); 17 CN IV (5l, 12r); 35 CN VI (15l, 20r) | 0.1-4V | 200 | 8.6-9.6 | NS | NS | NS | NS | 1 conjunctival hemorrhage, 1 periorbital ecchimosis, 1 corneal abrasion, ptosis, upper eyelid edema | conjunctival hemorrhage resolved, upper corneal abrasion, ptosis, upper eyelid edema improving |
| Jimenez et al 2024 [24] | 3 Chol. Gran., 3 Chond., 24 Chord., 20 PitNETs; 6 Men.  35 CS procedures | 56  (27M; 29F) | NS | t-EMG  f-EMG | intramuscular needle 13 mm | CN VI: LR | NS | 56 CN VI | 0.5-2V | NS | NS |  | 0 | NS | 46,4%  26 patients | NS | Resolved (n=14),  partial palsy (n=6), complete palsy (n=6) |

**Supplementary Table 2**

Summary of the studies included in the scoping review.

Abbreviations: Bipol, Bipolar; Chond., Chondrosarcoma; Chol. Gran., Cholesterol Granuloma; Chord., Chordomas; Craniop., Craniopharyngioma; CS, Cavernous Sinus; F, Female; f-EMG, free-run Electromyography; GTR, Gross Total Resection; IR, Inferior Rectus; l, left; LR, Lateral Rectus; M, Male; Men. Meningioma; Mono, Monopolar; MR, Medial Rectus; NS, Not specified; PitNET, Pituitary NeuroEndocrine Tumor; PR, Partial Resection; pt, patient; r, Right; Schw., Schwannoma; SOM, Superior Oblique Muscle; SR, Superior Rectus; V, Volt; t-EMG, trigger electromyography; ^ç^, Number and side of nerve monitoring not specified; *, the type of IONM is deducted from the description of the article; §, patients monitored.
