# Supplementary material for: Neurophysiological monitoring of cranial nerves III and VI in endoscopic skull base surgery: scoping review and comparative study
Source: Front Oncol. 2026 Sep 14;16:1831926. doi: 10.3389/fonc.2026.1831926 (PMC13616701; doi:10.3389/fonc.2026.1831926)
Supplement: Supplementary file 3 [file Table3.docx]

Supplementary Material

**Supplementary Table 3**

A summary of the advantages, disadvantages, and complications for each type of IONM electrode.

|  | **Advantages** | **Disadvantages** | **Complications** |
| --- | --- | --- | --- |
| **Surface Electrodes** | Non-invasive technique. Electrodes placement is quick and not extensive training is required. | High impedences value and signal to noise ratio. | Skin irritation; artifact-related false signals. |
| **Hookwire Electrodes** | Mini-invasive placement. | Complex insertion manouver. Extension cable is needed. Steeper learning courve. High impedences value and signal to noise ratio. High risk of displacement. | Artifact-related false signals. Local trauma (hematoma). |
| **Curved Needle Electrodes** | Stable placement. Very low impedence value and signal to noise ratio. | Invasive. Steeper learning courve. | Periorbital hematoma, infection, local tissue injury. |
