# Supplementary material for: Neurophysiological monitoring of cranial nerves III and VI in endoscopic skull base surgery: scoping review and comparative study
Source: Front Oncol. 2026 Sep 14;16:1831926. doi: 10.3389/fonc.2026.1831926 (PMC13616701; doi:10.3389/fonc.2026.1831926)
Supplement: Supplementary file 4 [file Table4.docx]

Supplementary Material

**Supplementary Table 4**

A summary comparing the signal characteristics for each different electrode types.

|  | **Spontaneous/manipulation-induced activities (fEMG)** | **Triggered muscolar activities (tEMG)** | **Corticobulbar evoked potentials (cbMEPs)** |
| --- | --- | --- | --- |
| **Surface Electrodes/subdermal needle recorded EOG** | Poorly interpretable signals due to overlapping sourrounding muscle activities. | Non-specific muscle activation detected. | Not recordable, except for one patient. |
| **Hookwire Electrodes (mono and bipolar registration)** | Difficult to identify muscular activity due to artifact. | Non-specific responses where recording was feasible. | Not recordable. |
| **Curved Needle Electrodes** | Clearly interpretable signals. | Feasible and specific except in case of electrodes displacement (2 patient). | Stable and reproducible response. |
